# Recent advances in cardiac magnetic resonance

**DOI:** 10.12688/f1000research.8383.1

**Published:** 2016-09-07

**Authors:** Simon Greulich, Andrew E. Arai, Udo Sechtem, Heiko Mahrholdt

**Affiliations:** 1 Division of Cardiology, Robert Bosch Medical Center, Stuttgart, Germany; 2 National Heart, Lung, and Blood Institute, National Institutes of Health, Department of Health and Human Services, Bethesda, MD, USA

**Keywords:** Late gadolinium enhancement, T2, T2w, mapping

## Abstract

Cardiac magnetic resonance (CMR) is a non-invasive imaging modality that has rapidly emerged during the last few years and has become a valuable, well-established clinical tool. Beside the evaluation of anatomy and function, CMR has its strengths in providing detailed non-invasive myocardial tissue characterization, for which it is considered the current diagnostic gold standard.

Late gadolinium enhancement (LGE), with its capability to detect necrosis and to separate ischemic from non-ischemic cardiomyopathies by distinct LGE patterns, offers unique clinical possibilities. The presence of LGE has also proven to be a good predictor of an adverse outcome in various studies.

T2-weighted (T2w) images, which are supposed to identify areas of edema and inflammation, are another CMR approach to tissue characterization. However, T2w images have not held their promise owing to several technical limitations and potential physiological concerns.

Newer mapping techniques may overcome some of these limitations: they assess quantitatively myocardial tissue properties in absolute terms and show promising results in studies for characterization of diffuse fibrosis (T1 mapping) and/or inflammatory processes (T2 mapping). However, these techniques are still research tools and are not part of the clinical routine yet.

T2* CMR has had significant impact in the management of thalassemia because it is possible to image the amount of iron in the heart and the liver, improving both diagnostic imaging and the management of patients with thalassemia.

CMR findings frequently have clinical impact on further patient management, and CMR seems to be cost effective in the clinical routine.

## Introduction

Although echocardiography is still the preferred initial cardiac imaging modality owing to its widespread distribution and low cost, the field of cardiovascular magnetic resonance (CMR) has emerged rapidly in the last few years. The advantages of CMR are lack of ionizing radiation, image quality that is not dependent on acoustic window, multi-planar imaging capability, and high spatial and temporal resolution, providing detailed information about myocardial anatomy, function, and tissue within a single exam as a one-stop shop.

In this manuscript, we will describe and discuss recent advances in the CMR assessment of heart disease, including the prognostic value of late gadolinium enhancement (LGE), advantages and limitations of T2-weighted (T2w) imaging, as well as of the newer mapping proton relaxation techniques (T1 native, T1 post contrast, and T2 mapping), and the benefit of T2* sequences in patients with thalassemia. Furthermore, we will present the most recent data on the cost effectiveness of CMR in the clinical routine.

## Late gadolinium enhancement

LGE images are acquired 10–20 minutes after the application of gadolinium-based contrast agents. At this point in time, most contrast has been washed out from normal myocardium (which is nulled, appearing black in the image), but in regions with enlarged extracellular/interstitial space due to acute necrosis, chronic scar, or fibrotic tissue, the contrast wash-out is altered, resulting in a hyper-intense signal (bright) in corresponding areas ^1^. Today, this robust technique is the most frequently applied and best validated method for myocardial tissue characterization. Recent data indicate that LGE is useful not only for diagnosis ^1, 2^ but also as a predictor of outcome ^3– 8^. The rare cases of nephrogenic systemic fibrosis (NSF) occurred only in patients with a glomerular filtration rate <30 mL/minute, in whom gadolinium is now considered to be contra-indicated ^9^.

**Figure 1.**
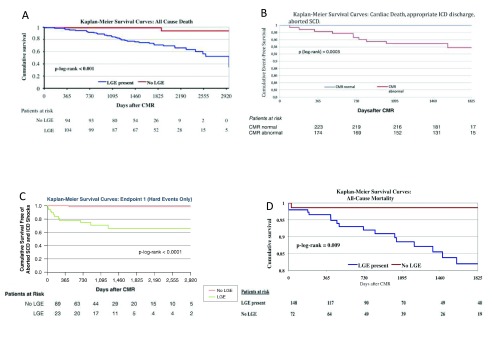
Kaplan Meier curves for late gadolinium enhancement (LGE)-positive and LGE-negative groups in patients with different cardiac diseases. Graphs **A** and **B** represent myocarditis, **C** represents sarcoidosis, and **D** represents hypertrophic cardiomyopathy (HCM). These graphs underline the high prognostic value of LGE–cardiac magnetic resonance (CMR). Patients who are LGE positive are at increased risk of suffering from adverse events, whereas patients who are LGE negative have an excellent prognosis. *Adapted from* 3– 5, 7 *with permission.* ICD, implantable cardioverter defibrillator; SCD, sudden cardiac death.

In patients with biopsy-proven viral myocarditis, the presence of LGE was the best independent predictor of all-cause mortality and of cardiac mortality ^3^. Conversely, patients with no LGE had an excellent prognosis. Furthermore, in a large population of more than 400 consecutive patients with suspected myocarditis (“all comers”), all patients with normal CMR (normal left ventricular [LV] ejection fraction, normal LV end-diastolic volume, and no LGE) had a good prognosis independent of their clinical symptoms and other findings ^4^ (see Figure 1).

Similar results were recently obtained in patients with sarcoidosis. In a cohort of 155 sarcoid patients, the presence of LGE was correlated with major adverse events such as ventricular tachycardia (VT) and sudden cardiac death (SCD) ^5^ ( Figure 1). In another study with sarcoid patients, LGE was quantified as percentage of LV mass and revealed the LGE burden as the best predictor of death/VT ^6^. For every 1% increase of LGE burden, the hazard of death/VT increased by 8%. This finding is an important recent advance, since establishing a critical threshold of LGE burden would facilitate risk stratification and may have a direct impact on patient management, e.g. indication for an implantable cardioverter defibrillator (ICD). However, more studies are needed to confirm these results.

LGE also provides prognostic information in hypertrophic cardiomyopathy (HCM), since the presence of LGE is a good independent predictor of all-cause and cardiac mortality in this population as well. In the LGE group, the odds ratio for all-cause mortality (death from any cause, including aborted SCD) was 5.47 and 8.01 for cardiac mortality. Of note, the presence of two classic clinical risk factors, which was a commonly employed criterion for ICD implantation in these patients, yielded an odds ratio of only 3.86 for all-cause mortality and 2.20 for cardiac mortality ^7^.

Nevertheless, the overall positive predictive value of LGE for major adverse cardiac events (MACE) is only moderate, whereas the negative predictive value of LGE seems to be excellent ( Figure 1). Therefore, it may be safe to conclude that a normal CMR is related to an excellent prognosis.

Despite this important prognostic information, we unfortunately cannot distinguish acute from chronic processes by using LGE. Thus, other CMR techniques such as T2w imaging were introduced. T2 is the time of transverse magnetization decay, which is prolonged in edematous, inflammatory myocardial tissue, appearing bright on T2w images ^10– 12^. As a major drawback, the image quality depends on a regular, norm-frequent heart rhythm and sufficient breath-hold during imaging acquisition. Unfortunately, this method is prone to signal artifacts, frequently resulting in non-diagnostic images, and seems not to hold its promise in the current clinical routine ^13, 14^.

Moreover, LGE has its strengths in detecting focal processes (e.g. infarcted myocardium vs. remote myocardium), whereas in diffuse processes the value of this technique is limited ^14^.

As a result of these limitations, the interest in new CMR techniques, such as T1 and T2 mapping, which provide absolute quantitative values rather than just visual or semi-quantitative interpretation of the images obtained, has been growing recently. Currently, most mapping data are available for the detection of diffuse fibrosis and/or inflammation. However, new studies even suggest a possible prognostic value similar or even superior to LGE ^15, 16^.

## T1 mapping

Native T1 mapping techniques represent an important recent advance in the non-invasive assessment of myocardial inflammation, since native myocardial T1 is prolonged by excess free water content ^17^. Post contrast T1 mapping techniques are able to determine the expansion of interstitial space/fibrosis (extracellular volume [ECV]) and separate it from replacement fibrosis (such as scarred myocardium) ^18, 19^. ECV allows estimation of the extracellular matrix in quantitative terms ^20^ by performing T1 mapping before and after contrast administration, assessing the volume of distribution of gadolinium. Thus, non-ischemic patterns of myocardial damage can be detected by T1 mapping in acute myocarditis ^21^. Moreover, T1 mapping recently expanded current knowledge by identifying additional myocardial abnormalities in comparison to conventional LGE, since T1 values can be prolonged in apparently normal regions without any LGE ^21, 22^. This potential role of T1 mapping in detecting diffusely diseased myocardium (not detectable by LGE) was confirmed by other studies dealing with aortic stenosis ^23^, systemic sclerosis ^24^, and systemic lupus erythematosus ^25^. However, whether these diffuse myocardial T1 abnormalities correspond to histologic findings, whether they are potentially reversible by an adequate treatment regimen, and whether they precede irreversible myocardial fibrosis as displayed by LGE need to be further investigated.

Nevertheless, two recent large studies indicate a potential prognostic impact of T1 mapping. Puntmann *et al*. prospectively enrolled 637 patients with dilated cardiomyopathy (DCM) who underwent CMR including T1 mapping and LGE ^15^. The primary endpoint was all-cause mortality, and the secondary endpoint was a composite of heart failure, mortality, and hospitalization. During follow-up, both T1 mapping techniques (native T1 and ECV), as well as LGE, were predictive of all-cause mortality and heart failure. Interestingly, in multivariable analyses, native T1 mapping was the sole independent predictor of all-cause and heart failure composite endpoints.

In addition, Schelbert *et al*. performed ECV analysis and LGE in 1172 patients, comparing the imaging results to the clinical outcome. They found that myocardial fibrosis measured by ECV was more strongly associated with outcomes (hospitalization for heart failure and/or death) than any myocardial damage detected by LGE ^16^.

## T2 mapping

T2 maps are generated by accumulating multiple images with different T2w, providing several points along the T2 decay curve yielding to direct quantification of the myocardial T2. T2 mapping seems to overcome the already-described severe limitations ^13, 14^ of the standard T2w sequences currently used for characterizing myocardial inflammation or salvage and may have a sensitivity and specificity of more than 90% for detecting acute myocarditis or Takotsubo cardiomyopathy ^26^.

T2 mapping also holds promise for sarcoid patients. In a recent study, 11 of 27 (41%) LGE-negative patients demonstrated an abnormal T2 map, and 7 of 23 patients with normal T2 mapping were LGE positive, suggesting a synergistic rather than a summative diagnostic value from both techniques to detect cardiac involvement in sarcoidosis ^27^.

## Limitations of mapping

Despite these promising results, there are several limitations with regard to mapping techniques:

1) Most centers use individual acquisition sequences; thus, values are not comparable between different centers and patient groups

2) There is no gold standard on how to analyze the maps, e.g. analysis of a single midventricular slice vs. a whole stack of myocardial slices displaying the whole ventricle vs. drawing a region of interest located in the interventricular septum. Diffuse myocardial measurements make sense on some pathological processes such as diffuse fibrosis when there is no localized abnormality on the map or other images. Localized measurements might make more sense than sectors or whole slice measurements if there was a clinical clue to a localized abnormality such as a regional wall motion abnormality

3) For analysis, care should be taken not to draw regions too close to the cavity of the left ventricle or the epicardium, since this partial volume effect would result in incorrect values

4) Most available data demonstrate an overlap of values between patients with cardiac diseases and healthy controls, lowering the validity of the technique

Although some of these points were addressed in a Society for CMR (SCMR) consensus paper, others were not clearly defined ^28^. However, a novel statement is likely to be revised in the next year or two, as the field has been rapidly evolving.

## Benefit of T2* sequences

CMR has had significant impact on the management of thalassemia because it is possible to image the amount of iron in the heart and the liver. Thalassemia is an inherited hemolytic anemia characterized by abnormal hemoglobin production but is particularly prevalent in the Middle East, West Africa, and the South Pacific. Alpha (α)-thalassemia may be the most common single gene disorder in the world, but several hundred other mutations can cause variants of thalassemia. Starting in the 1960’s, transfusions improved symptoms but led to iron overload. Around 1982, chelation therapy with desferrioxamine improved the prognosis of patients with thalassemia. Since 1999, mortality from heart failure in patients with thalassemia improved markedly, an improvement attributed to better identification and better guidance of chelation therapy made possible by T2*w CMR ^29^.

T2* CMR was the first of the quantitative measurements of relaxation properties to have significant clinical impact. In simple terms, T2* is primarily affected by local variations in the homogeneity of the magnetic field. Intramyocardial iron distorts the magnetic field and therefore reduces T2*. Practical measurements of cardiac T2* were developed around 1999 ^30^. T2* has been validated against human myocardial iron content ^31^. This is important because cardiac T2* predicts the likelihood that patients with thalassemia develop heart failure and arrhythmias ^32^. More importantly, cardiac T2* was a better predictor than liver iron content or serum ferritin levels. The latter observation may be explained by the slower clearance of iron from the heart than the liver ^33^. Thus, quantitative T2* CMR methods have improved both diagnostic imaging and the management of patients with thalassemia.

## Cost effectiveness of CMR

**Figure 2.**
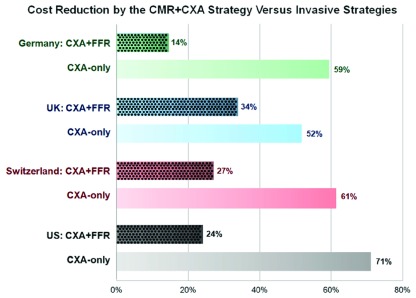
Percentage of cost reductions of the cardiac magnetic resonance (CMR)–X-ray coronary angiography (CXA) strategy vs. CXA + fractional flow reserve (FFR) and CXA-only strategy in patients with suspected coronary artery disease (CAD) analyzed for different healthcare systems (Germany, United Kingdom [UK], Switzerland, and United States [US]). CMR can help to minimize costs for the health system. *Adapted from* 36 *with permission*.

Despite its advantages, CMR was repeatedly criticized as a rather expensive and thus supplementary technique, which is not affordable in everyday clinical practice. To date, only a few studies have investigated the relationship between the diagnostic performance of CMR and costs for the healthcare system ^34, 35^. A recent report from the EuroCMR-Registry investigators suggests that CMR can be cost effective in daily clinical routine: in patients with suspected coronary artery disease (CAD), costs were calculated for diagnostic examinations (stress CMR, X-ray coronary angiography [CXA] with or without determination of the fractional flow reserve [FFR] to determine hemodynamic significant stenosis), revascularizations, and complications during a 1-year follow-up in more than 3600 patients from the EuroCMR-Registry ^36^. Patients were divided into three groups: 1) ischemia-positive patients on stress CMR underwent CXA and (potential) revascularization at the discretion of the treating physician; 2) in the second, hypothetical invasive group, costs were calculated for an initial CXA and additional FFR assessment in all vessels with ≥50% stenosis, and the same proportion of revascularizations and complications were applied as in the first group; and 3) in the CXA-only strategy, costs were calculated for CXA and for revascularization procedures of all ≥50% stenosis. The analysis revealed that a stress CMR and CXA strategy for patients with suspected CAD (first group) provides substantial cost reduction compared to patients undergoing CXA and FFR (second group) as well as CXA only (third group) in patients with low to intermediate disease prevalence (see Figure 2).

## Conclusion

In summary, LGE is the most validated tool for the evaluation of myocardial tissue with respect to diagnosis and prognosis but is limited in a) distinguishing acute processes from chronic processes and b) the detection of diffuse fibrosis.

Novel quantitative mapping techniques (T1 and T2 mapping) show promise, as they can detect diffuse fibrosis and myocardial edema and even demonstrate some prognostic impact, pointing towards a synergistic rather than a summative value compared to LGE alone. However, standardization of mapping sequences and establishment of normal values, which would make patients’ results comparable between different centers, are warranted and highly desired. Large prospective multicenter studies should further evaluate the role of their clinical utility before mapping techniques can be implemented in daily clinical practice. T2* is already ready for clinical routine, improving both diagnostic imaging and the management of patients with thalassemia.

Other CMR techniques, such as stress CMR for risk stratification in suspected CAD, have already been proven to be not only diagnostic and of prognostic relevance but also cost effective in the clinical routine.

## Abbreviations

CAD, coronary artery disease; CMR, cardiac magnetic resonance; CXA, X-ray coronary angiography; ECV, extracellular volume; FFR, fractional flow reserve; ICD, implantable cardioverter defibrillator; LGE, late gadolinium enhancement; LV, left ventricular; SCD, sudden cardiac death; T2w, T2-weighted; VT, ventricular tachycardia.

## References

[1] Mahrholdt H Wagner A Judd RM: Delayed enhancement cardiovascular magnetic resonance assessment of non-ischaemic cardiomyopathies. *Eur Heart J.* 2005;26(15):1461–74. 10.1093/eurheartj/ehi258 15831557

[2] Banka P Robinson JD Uppu SC: Cardiovascular magnetic resonance techniques and findings in children with myocarditis: a multicenter retrospective study. *J Cardiovasc Magn Reson.* 2015;17:96. 10.1186/s12968-015-0201-6 26576638 PMC4650339

[3] Grün S Schumm J Greulich S: Long-term follow-up of biopsy-proven viral myocarditis: predictors of mortality and incomplete recovery. *J Am Coll Cardiol.* 2012;59(18):1604–15. 10.1016/j.jacc.2012.01.007 22365425

[4] Schumm J Greulich S Wagner A: Cardiovascular magnetic resonance risk stratification in patients with clinically suspected myocarditis. *J Cardiovasc Magn Reson.* 2014;16(1):14. 10.1186/1532-429X-16-14 24461053 PMC3913958

[5] Greulich S Deluigi CC Gloekler S: CMR imaging predicts death and other adverse events in suspected cardiac sarcoidosis. *JACC Cardiovasc Imaging.* 2013;6(4):501–11. 10.1016/j.jcmg.2012.10.021 23498675

[6] Murtagh G Laffin LJ Beshai JF: Prognosis of Myocardial Damage in Sarcoidosis Patients With Preserved Left Ventricular Ejection Fraction: Risk Stratification Using Cardiovascular Magnetic Resonance. *Circ Cardiovasc Imaging.* 2016;9(1):e003738. 10.1161/CIRCIMAGING.115.003738 26763280 PMC4718184

[7] Bruder O Wagner A Jensen CJ: Myocardial scar visualized by cardiovascular magnetic resonance imaging predicts major adverse events in patients with hypertrophic cardiomyopathy. *J Am Coll Cardiol.* 2010;56(11):875–87. 10.1016/j.jacc.2010.05.007 20667520

[8] Cheong BY Muthupillai R Wilson JM: Prognostic significance of delayed-enhancement magnetic resonance imaging: survival of 857 patients with and without left ventricular dysfunction. *Circulation.* 2009;120(21):2069–76. 10.1161/CIRCULATIONAHA.109.852517 19901193

[9] Beckett KR Moriarity AK Langer JM: Safe Use of Contrast Media: What the Radiologist Needs to Know. *Radiographics.* 2015;35(6):1738–50. 10.1148/rg.2015150033 26466182

[10] Aletras AH Tilak GS Natanzon A: Retrospective determination of the area at risk for reperfused acute myocardial infarction with T2-weighted cardiac magnetic resonance imaging: histopathological and displacement encoding with stimulated echoes (DENSE) functional validations. *Circulation.* 2006;113(15):1865–70. 10.1161/CIRCULATIONAHA.105.576025 16606793

[11] Fernández-Jiménez R Sánchez-González J Agüero J: Myocardial edema after ischemia/reperfusion is not stable and follows a bimodal pattern: imaging and histological tissue characterization. *J Am Coll Cardiol.* 2015;65(4):315–23. 10.1016/j.jacc.2014.11.004 25460833

[12] Nordlund D Klug G Heiberg E: Multi-vendor, multicentre comparison of contrast-enhanced SSFP and T2-STIR CMR for determining myocardium at risk in ST-elevation myocardial infarction. *Eur Heart J Cardiovasc Imaging.* 2016;17(7):744–53. 10.1093/ehjci/jew027 27002140 PMC4907382

[13] Kim HW van Assche L Jennings RB: Relationship of T2-Weighted MRI Myocardial Hyperintensity and the Ischemic Area-At-Risk. *Circ Res.* 2015;117(3):254–65. 10.1161/CIRCRESAHA.117.305771 25972514 PMC4503326

[14] Greulich S Ferreira VM Dall'Armellina E: Myocardial Inflammation–Are We There Yet? *Curr Cardiovasc Imaging Rep.* 2015;8(3):6. 10.1007/s12410-015-9320-6 25705323 PMC4330458

[15] Puntmann VO Carr-White G Jabbour A: T1-Mapping and Outcome in Nonischemic Cardiomyopathy: All-Cause Mortality and Heart Failure. *JACC Cardiovasc Imaging.* 2016;9(1):40–50. 10.1016/j.jcmg.2015.12.001 26762873

[16] Schelbert EB Piehler KM Zareba KM: Myocardial Fibrosis Quantified by Extracellular Volume Is Associated With Subsequent Hospitalization for Heart Failure, Death, or Both Across the Spectrum of Ejection Fraction and Heart Failure Stage. *J Am Heart Assoc.* 2015;4(12): pii: e002613. 10.1161/JAHA.115.002613 26683218 PMC4845263

[17] Higgins CB Herfkens R Lipton MJ: Nuclear magnetic resonance imaging of acute myocardial infarction in dogs: alterations in magnetic relaxation times. *Am J Cardiol.* 1983;52(1):184–8. 10.1016/0002-9149(83)90093-0 6858909

[18] Kehr E Sono M Chugh SS: Gadolinium-enhanced magnetic resonance imaging for detection and quantification of fibrosis in human myocardium *in vitro*. *Int J Cardiovasc Imaging.* 2008;24(1):61–8. 10.1007/s10554-007-9223-y 17429755

[19] Jerosch-Herold M Sheridan DC Kushner JD: Cardiac magnetic resonance imaging of myocardial contrast uptake and blood flow in patients affected with idiopathic or familial dilated cardiomyopathy. *Am J Physiol Heart Circ Physiol.* 2008;295(3):H1234–H1242. 10.1152/ajpheart.00429.2008 18660445 PMC2544489

[20] Kellman P Wilson JR Xue H: Extracellular volume fraction mapping in the myocardium, part 1: evaluation of an automated method. *J Cardiovasc Magn Reson.* 2012;14:63. 10.1186/1532-429X-14-63 22963517 PMC3441905

[21] Ferreira VM Piechnik SK Dall'Armellina E: Native T1-mapping detects the location, extent and patterns of acute myocarditis without the need for gadolinium contrast agents. *J Cardiovasc Magn Reson.* 2014;16:36. 10.1186/1532-429X-16-36 24886708 PMC4041901

[22] Puntmann VO Voigt T Chen Z: Native T1 mapping in differentiation of normal myocardium from diffuse disease in hypertrophic and dilated cardiomyopathy. *JACC Cardiovasc Imaging.* 2013;6(4):475–84. 10.1016/j.jcmg.2012.08.019 23498674

[23] Bull S White SK Piechnik SK: Human non-contrast T1 values and correlation with histology in diffuse fibrosis. *Heart.* 2013;99(13):932–7. 10.1136/heartjnl-2012-303052 23349348 PMC3686317

[24] Ntusi NA Piechnik SK Francis JM: Subclinical myocardial inflammation and diffuse fibrosis are common in systemic sclerosis--a clinical study using myocardial T1-mapping and extracellular volume quantification. *J Cardiovasc Magn Reson.* 2014;16(1):21. 10.1186/1532-429X-16-21 24593856 PMC3996013

[25] Puntmann VO D'Cruz D Smith Z: Native myocardial T1 mapping by cardiovascular magnetic resonance imaging in subclinical cardiomyopathy in patients with systemic lupus erythematosus. *Circ Cardiovasc Imaging.* 2013;6(2):295–301. 10.1161/CIRCIMAGING.112.000151 23403334

[26] Thavendiranathan P Walls M Giri S: Improved detection of myocardial involvement in acute inflammatory cardiomyopathies using T2 mapping. *Circ Cardiovasc Imaging.* 2012;5(1):102–10. 10.1161/CIRCIMAGING.111.967836 22038988 PMC3261300

[27] Crouser ED Ono C Tran T: Improved detection of cardiac sarcoidosis using magnetic resonance with myocardial T2 mapping. *Am J Respir Crit Care Med.* 2014;189(1):109–12. 24381994 10.1164/rccm.201309-1668LE PMC3919128

[28] Moon JC Messroghli DR Kellman P: Myocardial T1 mapping and extracellular volume quantification: a Society for Cardiovascular Magnetic Resonance (SCMR) and CMR Working Group of the European Society of Cardiology consensus statement. *J Cardiovasc Magn Reson.* 2013;15(1):92. 10.1186/1532-429X-15-92 24124732 PMC3854458

[29] Modell B Khan M Darlison M: Improved survival of thalassaemia major in the UK and relation to T2* cardiovascular magnetic resonance. *J Cardiovasc Magn Reson.* 2008;10(1):42. 10.1186/1532-429X-10-42 18817553 PMC2563008

[30] Anderson LJ Holden S Davis B: Cardiovascular T2-star (T2*) magnetic resonance for the early diagnosis of myocardial iron overload. *Eur Heart J.* 2001;22(23):2171–9. 10.1053/euhj.2001.2822 11913479

[31] Carpenter JP He T Kirk P: On T2* magnetic resonance and cardiac iron. *Circulation.* 2011;123(14):1519–28. 10.1161/CIRCULATIONAHA.110.007641 21444881 PMC3435874

[32] Kirk P Roughton M Porter JB: Cardiac T2* magnetic resonance for prediction of cardiac complications in thalassemia major. *Circulation.* 2009;120(20):1961–8. 10.1161/CIRCULATIONAHA.109.874487 19801505 PMC2784198

[33] Anderson LJ Westwood MA Holden S: Myocardial iron clearance during reversal of siderotic cardiomyopathy with intravenous desferrioxamine: a prospective study using T2* cardiovascular magnetic resonance. *Br J Haematol.* 2004;127(3):348–55. 10.1111/j.1365-2141.2004.05202.x 15491298

[34] Miller CD Hwang W Hoekstra JW: Stress cardiac magnetic resonance imaging with observation unit care reduces cost for patients with emergent chest pain: a randomized trial. *Ann Emerg Med.* 2010;56(3):209–219.e2. 10.1016/j.annemergmed.2010.04.009 20554078 PMC3716462

[35] Petrov G Kelle S Fleck E: Incremental cost-effectiveness of dobutamine stress cardiac magnetic resonance imaging in patients at intermediate risk for coronary artery disease. *Clin Res Cardiol.* 2015;104(5):401–9. 10.1007/s00392-014-0793-0 25395355 PMC4544498

[36] Moschetti K Petersen SE Pilz G: Cost-minimization analysis of three decision strategies for cardiac revascularization: results of the "suspected CAD" cohort of the european cardiovascular magnetic resonance registry. *J Cardiovasc Magn Reson.* 2016;18:3. 10.1186/s12968-015-0222-1 26754743 PMC4709988

